# Critically buried avalanche victims can develop severe hypothermia in less than 60 min

**DOI:** 10.1186/s13049-023-01092-y

**Published:** 2023-06-15

**Authors:** Simon Rauch, Julia Kompatscher, Andreas Clara, Iris Öttl, Giacomo Strapazzon, Marc Kaufmann

**Affiliations:** 1 Institute of Mountain Emergency Medicine, Eurac Research, Via Ipazia 2, Bolzano, 39100 Italy; 2 Department of Anaesthesia and Intensive Care Medicine, Hospital of Merano, Via Rossini 5, Merano, 39012 Italy; 3 Aiut Alpin Dolomites Helicopter Emergency Medical Service, Pontives 24, Laion, 39040 Italy; 4 Department of Emergency Medicine, Anaesthesia and Intensive Care Medicine, Hospital of Bolzano, Via Lorenz Böhler, 5, Bolzano, 39100 Italy; 5 Corpo Nazionale Soccorso Alpino e Speleologico - CNSAS, Milano, Italy

**Keywords:** Avalanche, Avalanche burial, Accidental hypothermia, Extracorporeal rewarming, HOPE score, Organ donation

## Abstract

**Background:**

A major challenge in the management of avalanche victims in cardiac arrest is differentiating hypothermic from non-hypothermic cardiac arrest, as management and prognosis differ. Duration of burial with a cutoff of 60 min is currently recommended by the resuscitation guidelines as a parameter to aid in this differentiation However, the fastest cooling rate under the snow reported so far is 9.4 °C per hour, suggesting that it would take 45 min to cool below 30 °C, which is the temperature threshold below which a hypothermic cardiac arrest can occur.

**Case presentation:**

We describe a case with a cooling rate of 14 °C per hour, assessed on site with an oesophageal temperature probe. This is by far the most rapid cooling rate after critical avalanche burial reported in the literature and further challenges the recommended 60 min threshold for triage decisions. The patient was transported under continuous mechanical CPR to an ECLS facility and rewarmed with VA-ECMO, although his HOPE score was 3% only. After three days he developed brain death and became an organ donor.

**Conclusions:**

With this case we would like to underline three important aspects: first, whenever possible, core body temperature should be used instead of burial duration to make triage decisions. Second, the HOPE score, which is not well validated for avalanche victims, had a good discriminatory ability in our case. Third, although extracorporeal rewarming was futile for the patient, he donated his organs. Thus, even if the probability of survival of a hypothermic avalanche patient is low based on the HOPE score, ECLS should not be withheld by default and the possibility of organ donation should be considered.

## Background

Differentiating hypothermic from non-hypothermic (i.e., asphyctic or traumatic) cardiac arrest is a major challenge in the management of avalanche victims, as treatment and prognosis differ. The core body temperature (CBT) threshold below which a hypothermic cardiac arrest can occur in young and healthy persons is 30 °C [1]. Cooling rate after critical avalanche burial is variable [2] with a maximal cooling rate reported in the literature of 9.4 °C per hour [3, 4]. Assuming a pre-burial CBT of 37 °C it would therefore take 45 min to decrease CBT to < 30 °C after critical avalanche burial. The current resuscitation guidelines [1] recommend burial duration with a cutoff of 60 min as a parameter to aid in the differentiation between hypothermic and non-hypothermic cardiac arrest. Although practical to use and remember, burial duration with the 60 min cutoff does not allow to exclude hypothermia as a possible cause of cardiac arrest.

Here we describe a case with a very fast cooling rate after critical avalanche burial, further challenging the 60 min threshold recommended for triage decisions [1].

## Case presentation

A 49-year-old male backcountry skier (height 171 cm, weight 72 kg, BMI 24.6 kg/m^2^) was caught in an avalanche during ascent at 2,400 m above sea level. He was lightly dressed (base layer and mid layer with open zip), ambient temperature was − 8 °C. Companions located the victim rapidly and after arrival of the mountain rescue service the patient was extricated from a burial depth of 2.5 m. After 45 min of burial the face and chest of the victim were exposed. The patient´s airway was patent but he had absent respiration and was pulseless, so cardiopulmonary resuscitation (CPR) with face mask ventilation and manual chest compressions was immediately commenced. Defibrillator pads were then attached, and the ECG showed asystole. Five minutes after CPR initiation the patient´s trachea was intubated, and an oesophageal temperature probe was placed under laryngoscopic view. CBT was 24.7 °C with only minimal fluctuations (+/- 0.2 °C). The patient was brought under continuous, mechanical CPR to hospital. Oesophageal temperature on arrival at hospital was 24.5 °C, which was confirmed by a bladder temperature measurement. Serum potassium was 7.8 mmol/l with blood drown from a central venous catheter. Thirty minutes after hospital admission and after a total of 100 min CPR, venoarterial (VA-)ECMO was started. At a CBT of 26 °C PEA appeared, at 29 °C ventricular fibrillation, and the patient was successfully defibrillated and weaned from VA-ECMO after 30 h. However, brain CT evidenced severe hypoxic brain damage and after 66 h from hospital admission brain death was diagnosed. The patient donated his kidneys, liver, and lungs.

## Discussion and conclusions

With this case we would like to point out three aspects. First, the reported cooling rate is the most rapid documented in the literature [3, 4]. Assuming a baseline CBT of 37 °C, the patient cooled by approximately 12 °C in 50 min, corresponding to about 14 °C in one hour when assuming a linear decrease. The CBT was measured with an oesophageal temperature probe which is considered the gold standard measurement technique in out-of-hospital patients with a secured airway [5, 6]. The temperature probe was placed a few minutes after unburial of the patient, therefore post-extrication cooling can be considered minimal, also because the patient was in cardiac arrest and cooling rate might be higher in victims with cardiac output compared to victims in cardiac arrest [7]. This is important as previous studies have shown that the cooling rate during burial may be lower than after extrication [2, 8] and triage decisions should exclude post-extrication cooling [9]. The cooling rates during snow burial reported in the literature vary widely from 0.1 °C/h to 9.4 °C/h [2, 4, 10, 11]. The European Resuscitation Council (ERC) guidelines 2021 recommend that avalanche victims with out-of-hospital cardiac arrest and duration of burial < 60 min should be managed like normothermic patients [1]. Interestingly, while in the ERC guidelines 2015 the measurement of a CBT was recommended during CPR also in case of a burial duration < 60 min [12], this is no longer explicitly mentioned in the 2021 guidelines [1]. Only the recent recommendations of the International Commission of Mountain Emergency Medicine recommend oesophageal temperature measurement also if burial duration is < 60 min and to use CBT over burial duration for triage decisions [13]. The very rapid cooling rate in our case corroborates this. Though asphyxia remains the main cause for cardiac arrest after a burial duration of < 60 min, our case shows that severe hypothermia can occur even within the first hour after critical avalanche burial. Several factors might have increased heat loss and contributed to the very rapid cooling rate in our case, e.g., sweating and thin garments worn during ascent. Also, hypercapnia, which may develop in critically buried avalanche victims, speeds up cooling by causing systemic vasodilation. Although asystole in an unwitnessed cardiac arrest after avalanche burial is associated with a poor outcome [14], the decision to extracorporeally rewarm our patient was made because his airway was patent, his CBT was far below 30 °C and he did not show any signs of major trauma.

The second important aspect of this case is about prognostication. Guidelines [1, 13] recommend that in-hospital prognostication of successful rewarming should include estimation of the survival probability using the HOPE score [15, 16]. While the HOPE score is well validated in hypothermic, non-avalanche patients [15], the evidence for its discriminatory ability in hypothermic avalanche patients is poor [17]. Yet, the HOPE score is better validated than the classical prognostication and triage model based on serum potassium and CBT [18]. The HOPE score in our case was 3% and therefore far below the proposed threshold of 10%. Given the unfavorable outcome in our case, the HOPE score and the proposed cutoff had a good discriminatory ability.

Yet, extracorporeal rewarming in our case was not futile. Although the patient did not survive, four organs were explanted and successfully transplanted. In times of organ shortage, this aspect should not be underestimated. In a recent single-center study by Métrailler-Mermoud [19], seven out of 66 (11%) avalanche victims became organ donors and organs from brain-dead avalanche victims have been transplanted with good initial and long-term graft function [20]. Thus, even if the probability of survival of a hypothermic avalanche patient is low based on the HOPE score and asphyctic cardiac arrest more likely, extracorporeal life support should not be withheld by default and the possibility of organ donation should be considered.

## Data Availability

The detailed data regarding the case presented are available from the corresponding author on reasonable request.

## List of abbreviations

CBT: core body temperature
CT: computed tomography
CPR: cardiopulmonary resuscitation
ECLS: extracorporeal life support
VA-ECMO: venoarterial extracorporeal membrane oxygenation
